# The efficacy and safety of combined administration of intravenous and topical tranexamic acid in primary total knee arthroplasty: a meta-analysis of randomized controlled trials

**DOI:** 10.1186/s12891-018-2181-9

**Published:** 2018-09-07

**Authors:** Huazhang Xiong, Yi Liu, Yi Zeng, Yuangang Wu, Bin Shen

**Affiliations:** 1 Department of Orthopaedic Surgery, West China Hospital, West China Medical School, Sichuan University, Chengdu, Sichuan Province 610041 People’s Republic of China; 2 Department of Orthopedic Surgery, The First Affiliated Hospital of Zunyi Medical College, Zunyi, 563003 Guizhou Province China

**Keywords:** Total knee arthroplasty, Tranexamic acid, Intravenous, Topical, Blood loss

## Abstract

**Background:**

The combined administration of intravenous (IV) and topical tranexamic acid (TXA) in primary total knee (TKA) knee remains controversial. The purpose of this meta-analysis was to assess the efficacy and safety of combined administration of IV and topical TXA in primary TKA.

**Methods:**

PubMed, EMBASE, Cochrane Central Register of Controlled Trials, Web of Science, Google Search Engine and China National Knowledge Infrastructure databases were searched for randomized controlled trials (RCTs) were comparing the combined administration of IV and topical TXA following primary TKA. The primary outcomes were total blood loss, maximum hemoglobin drop, and deep venous thrombosis (DVT) and/or pulmonary embolism (PE). The second outcomes were drainage volume and transfusion requirements. Data were analyzed using RevMan 5.3.

**Results:**

A total of 6 RCTs involving 701 patients were included in the meta-analysis. The combined group provided lower total blood loss (MD − 156.34 mL, 95% CI, − 241.51 to − 71.18; *P* = 0.0003), drainage volume (MD − 43.54 mL, 95% CI, − 67.59 to − 19.48; *P* = 0.0004), maximum hemoglobin drop (MD − 0.56 g/dl, 95% CI, − 0.93 to − 0.19; *P* = 0.003) than IV TXA alone. No significant difference were found in terms of transfusion requirements (RR 0.48, 95% CI, 0.16 to 1.44; *P* = 0.19), DVT (RR 1.01, 95% CI, 0.14 to 7.12; *P* = 0.99) and PE (RR 0.33, 95% CI, 0.01 to 7.91; *P* = 0.49) between the two group. Subgroup analyses shows that the combined group was less total blood loss in non-tourniquet (*P* = 0.0008), topical TXA dose > 1.5 g (*P* <  0.00001) and number of IV TXA ≥ 2 doses (*P* = 0.005) of TXA compared with the IV group alone.

**Conclusions:**

The available evidence indicates combined group were associated with lower total blood loss, drainage volume, and maximum hemoglobin drop. A similar transfusion requirement was found in both groups. Subgroup analyses demonstrates that total blood loss was less in patients with non-tourniquet, topical TXA dose > 1.5 g and number of IV TXA ≥ 2 doses of TXA. There was no increase the rates of DVT and PE.

## Background

Total knee arthroplasty (TKA) and total hip arthroplasty (THA) is an effective orthopedic procedure for patients with severe knee and hip diseases [1–4]. However, significant blood loss may occur due to hyper-fibrinolysis induced by surgical trauma or tourniquet. Thus, it often leads to significant postoperative anemia and transfusion requirements [5, 6]. Postoperative anemia may be an important issue associated with adverse events, including increased mortality and morbidity and prolonged hospitalization due to transfusion-related needs.

Tranexamic acid (TXA) is a synthetic lysine analog, it can competitively inhibit the activation of plasminogen and plasmin binding protein [7, 8]. Several randomized controlled trials (RCTs) [9, 10] and meta-analysis studies [11, 12] have shown that intravenous (IV) [13, 14], topical (TA) [8] or oral [15] application of TXA can successfully reduce blood loss and transfusions in primary TKA without increasing the risk of thrombosis. Recently, an increasing number of studies have focused on the issue that when compared with only IV or TA TXA, whether combination application of IV and topical TXA has additional benefits in primary TKA [16, 17]. Compared with IV TXA, TA application has the advantage of being easy to administer, it leads to 70% lower systemic absorption and thus may be a safer alternative to giving it systemically. Additionally, topical application of TXA has the advantage of inducing partial microvascular hemostasis by stopping fibrin clot dissolution in the affected area [4, 8, 11, 18]. Once topically applied, TXA is rapidly absorbed and achieves the effect of hemostasis.

Several meta-analyses [19–21] were performed to evaluate the combination of IV and topical TXA in primary TKA. However, it may have some limitations and the conclusion might have the bias: First, as they included both TKA and THA in the analysis [19, 20], they did not account for the difference in the type of surgery. It cannot draw meaningful conclusions, and we believed that stricter criteria need to be applied to determine the benefits of combining TXA in a meta-analysis. Second, these studies were also affected by many other confounding factors [19–21], such as the application of tourniquet or non-tourniquet, and different topical TXA dose or the number of IV TXA. Thus, subgroup analysis based on the application of tourniquet or non-tourniquet, and topical TXA dose (≤1.5 g or > 1.5 g) or the number of IV TXA (single or ≥ 2 doses) were conducted, resulting in more accurate conclusions. Therefore, because of this bias factor, the efficacy of the combined IV and topical TXA in primary TKA has not been clearly concluded. Currently, there have been some well-designed studies [21–24] comparing the efficacy of combined administration of IV and topical TXA versus IV-TXA alone during TKA. Thus, the authors performed a meta-analysis to assess the highest evidence-based (level I) studies in order to investigate the effectiveness and safety of combined IV and TA application of TXA versus single IV TXA after primary TKA in regarding with (1) blood loss, including total blood loss and drainage volume; (2) transfusion requirements and maximum hemoglobin drop; and (3) thromboembolic complications, including deep venous thrombosis (DVT) and/or pulmonary embolism (PE). Additionally, subgroup analyses were also conducted to evaluate the benefits of the application of tourniquet or non-tourniquet, different topical (≤1.5 g or > 1.5 g) or the number of IV TXA (single or ≥ 2 doses) for total blood loss, maximum hemoglobin drop and transfusion requirements.

## Methods

The method used for this meta-analysis is based on the recommended PRISMA checklist guidelines [25]. The study was registered in the Research Registration Unique Identifying Number (review registry 249; http://www.researchregistry.com).

### Search strategy

We searched the following electronic databases: PubMed (1966 to December 2017), Embase (1974 to December 2017), Cochrane Central Register of Controlled Trials (December 2017) and Web of Science (1990 to December 2017). To identify additional potential studies, we also used the Google Search Engine (December 2017) and China National Knowledge Infrastructure (December 2017). We used the following keywords to search the database above: (total knee arthroplasty OR total knee replacement OR TKA OR TKR) AND (Tranexamic acid OR TXA OR TA). A search strategy with “PubMed” as an example in a manuscript: #1 Total Knee Arthroplasty; #2 Total Knee Replacement; #3 TKA; #4 TKR; #5 #1 OR #2 OR #3 OR #4; #6 Tranexamic Acid; #7 TXA; #8 TA; #9 #6 OR #7 OR #8; #10 #5 AND #9. There is no restriction on language and region.

### Inclusion criteria

The inclusion criteria for these studies were performed as follows: (1) studies were RCTs that included combined IV and topical application of TXA, and IV application of TXA; **(2) patients were performed primary unilateral TKA; and (3)** The outcomes of each RCTs included at least one of the following: blood loss, drainage volume, transfusion requirements, maximum hemoglobin drop, DVT, and PE. The studies were excluded if: (1) there were no sufficient outcomes; (2) revision **or simultaneous bilateral total knee arthroplasty. All titles and abstracts were** independently reviewed by two reviewers (XXX, XXX) to identify potential studies. These eligible studies were then obtained for inclusion based on the review of the full text. The differences were resolved by consensus after discussion, or a third reviewer was consulted if necessary.

### Assessment of methodological quality

Two reviewers (XXX, XXX) assessed independently the methodological quality as described by the Cochrane Collaboration for Systematic Reviews [26]. The six items included random sequence generation, allocation sequence concealment, blinding, incomplete outcome data, selective outcome reporting, and other risks. The overall methodological quality of each included study was characterized as low (low risk of bias), high (high risk of bias), and unclear (unclear risk of bias). Additionally, the two reviewers (XXX, XXX) used the modified Jadad scale to assess the risk of bias of the included studies [27]. Studies obtaining 4 or more points (up to 8 points) is considered to be of high quality, and the differences will be resolved by consensus after discussion, and if necessary, the third reviewer was consulted (XXX).

### Outcome measures

The effectiveness and safety of combined IV and TA application of TXA versus single IV TXA after primary TKA in this meta-analysis were compared. The primary outcomes were total blood loss, maximum hemoglobin drop, and deep venous thrombosis (DVT) and/or pulmonary embolism (PE). The second outcomes were drainage volume and transfusion requirements. Furthermore, Subgroup analysis was also performed based on whether the use of tourniquet and drainage tube to compare the additional benefits for blood loss.

### Data extraction

Two reviewers (XXX, XXX) independently extracted outcomes from the included studies. Their data includes authors, publication year, patients, age, and the intervention method of TXA, the method of DVT prophylaxis and screening, blood transfusion criterion. **If the study reported the same patient during the different follow-up period, we chose a longer follow-up time to avoid duplication of data.**

### Data synthesis

Statistical analyses of the meta-analysis were performed using RevMan 5 software (Version 5.3, the Cochrane Collaboration, UK). For continuous data, the mean differences (MD) and 95% confidence interval (CI) were calculated, such as total blood loss, drainage volume, and maximum hemoglobin drop. For dichotomous data, the risk ratio (RR) and 95% confidence interval (CI) were calculated, such as transfusion requirements, DVT or PE. The chi-squared test and I2 statistic were used to assess statistical heterogeneity. If the chi-squared test > 0.1 or the I^2^ < 50%, the fixed-effects model was chosen. Otherwise, a random-effects model was chosen. Publication bias was tested independently using funnel plots of total blood loss, drainage volume, maximum hemoglobin drop, transfusion requirements and DVT. If the funnel plot was symmetric, then there was a low potential for publication bias, or vice-versa.

## Results

### Search results

**Fig. 1:**
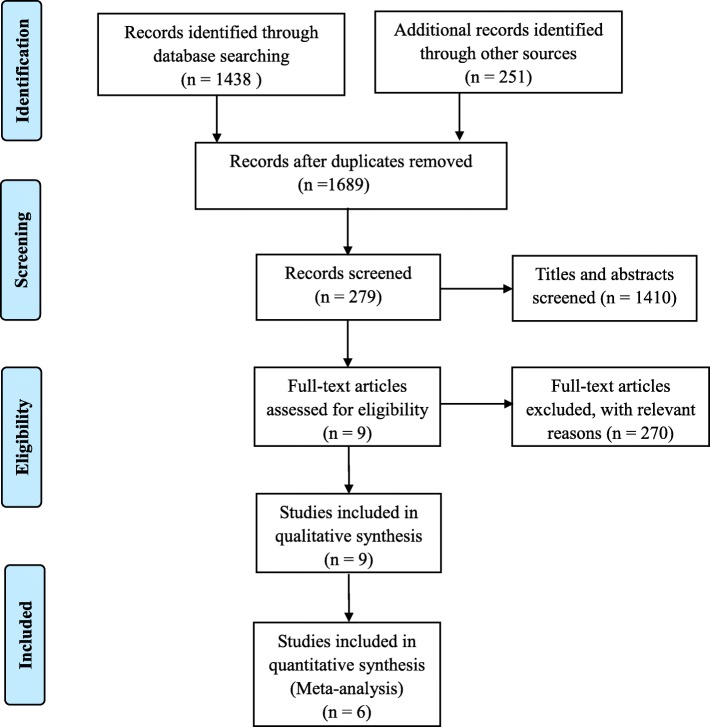
Flow chart of inclusion and exclusion for eligible studies

The flow chart in Fig. 1 shows the process by screening the potential studies. A total of 1689 studies were screened through the initial search, 1410 were excluded on the basis of their titles and abstracts, and then leaving 279 were read for full-text. After scanning full-text, 273 were also excluded since it did not meet inclusion criteria. Thus, 6 RCTs had been published between 2014 and 2017 used in the meta-analysis [16, 17, 22–24, 28]. These studies included 351 patients in combined administration of IV and topical TXA group (combined group) and 350patients in IV TXA group (IV TXA group). Sample size in included trials ranged from 25 to 95. Of all 6 studies, 5 studies were published in the English language [16, 17, 22–24], 1 study was published in the Chinese language [28]. Randomization was conducted in all 6 studies [16, 17, 22–24, 28]. All but 1 study [28] reported randomization method, of which 4 studies [17, 22–24] were reported using a computer-generated randomization, and in 1 [16] study reported the use of sealed envelope technique. There were 4 studies [16, 17, 22, 24] performed a clear blinding. All but two studies [17, 22] performed drainage, and the tourniquet was applied in four studies [16, 17, 23, 24]. All patients in the included studies received DVT prophylaxis of physical and chemical methods, including intermittent pneumatic compression, low-molecular-weight heparin, aspirin or rivaroxaban. Table 1 summarizes the baseline characteristics of included studies.

**Table 1:** Characteristics of included studies

Author	Diagnose	No. Of patients	Age (years)	Interventions	Drainage	Tourniquet	Transfusion criteria	DVT prophylaxis	DVT screen
Huang [16]	OA	VI: 92	VI: 64.7	VI: 3 g used before inflation	Yes	Yes	70 g/L ≤ Hb ≤ 90 g/L with anemia, Hb ≤70 g/L	LMWH Rivaroxaban	Ultrasound
		Combined: 92	Combined: 65.4	Combined:1.5 g used intravenously before inflation + 1.5 g used topically					
Jain [17]	OA	VI:60	VI: 70.0	VI: 15 mg/kg 30 min before skin incision, 10 mg/kg was repeated 3 and 6 h later.	No	No	7.0 g/d L ≤ Hb ≤ 8.0 g/dL with symptomatic anemia or Hb ≤7.0 g/dL	Aspirin	Clinical+ ultrasound
		Combined:59	Combined: 68.27	Combined: Same IV dose + 2 g used before closure of arthrotomy					
Nielsen[22]	NS	VI:30	VI: 63.2	VI: 1 g used + 100 mL of saline used after closure of the capsule	No	No	Hb < 7.5 g/dL or < 10 g/dL with heart disease or Hb reduced > 25%	Rivaroxaban	Clinical
		Combined:30	Combined: 65.5	Combined: same IV dose+ 3 g used after closure of the capsule					
Lee [23]	OA	VI:93	VI: 73.4	VI: 10 mg/kg 30 min before deflation, same dose repeated 3 h later	Yes	Yes	7.0 g/dL ≤ Hb ≤ 8.0 g/dL with symptomatic anemia or Hb ≤7.0 g/dL	Rivaroxaban, Aspirin	Clinical +CT
		Combined:95	Combined: 72.1	Combined: 10 mg/kg 30 min before deflation, same dose repeated 3 h later + 2 g injected.					
Song [24]	OA	VI:50	VI: 69.2	VI: 10 mg/kg 20 min before inflation,10 mg/kg 15 min before deflation, and 10 mg/kg 3 h later.	Yes	Yes	Hb < 8 g/%	LMWH	Ultrasound + CT
		Combined:50	Combined: 70.8	Combined: Same IV dose + 1.5 g used topically after wound closure					
Liu [19]	OA	VI:25	VI: NS	VI: 1 g 10 min before inflation	Yes	Yes	Hb ≤8 g/dL	Rivaroxaban	Ultrasound
		Combined:25	Combined: NS	Combined: same VI dose + 1 g 10 min before inflation					

Abbreviation: *OA* osteoarthritis, *VI* intravenous, ***NS*** **not stated,** *DVT* deep venous thrombosis, *Hb* hemoglobin, *LMWH* low-molecular weight heparin, *CT* computed tomographic

**Fig. 2:**
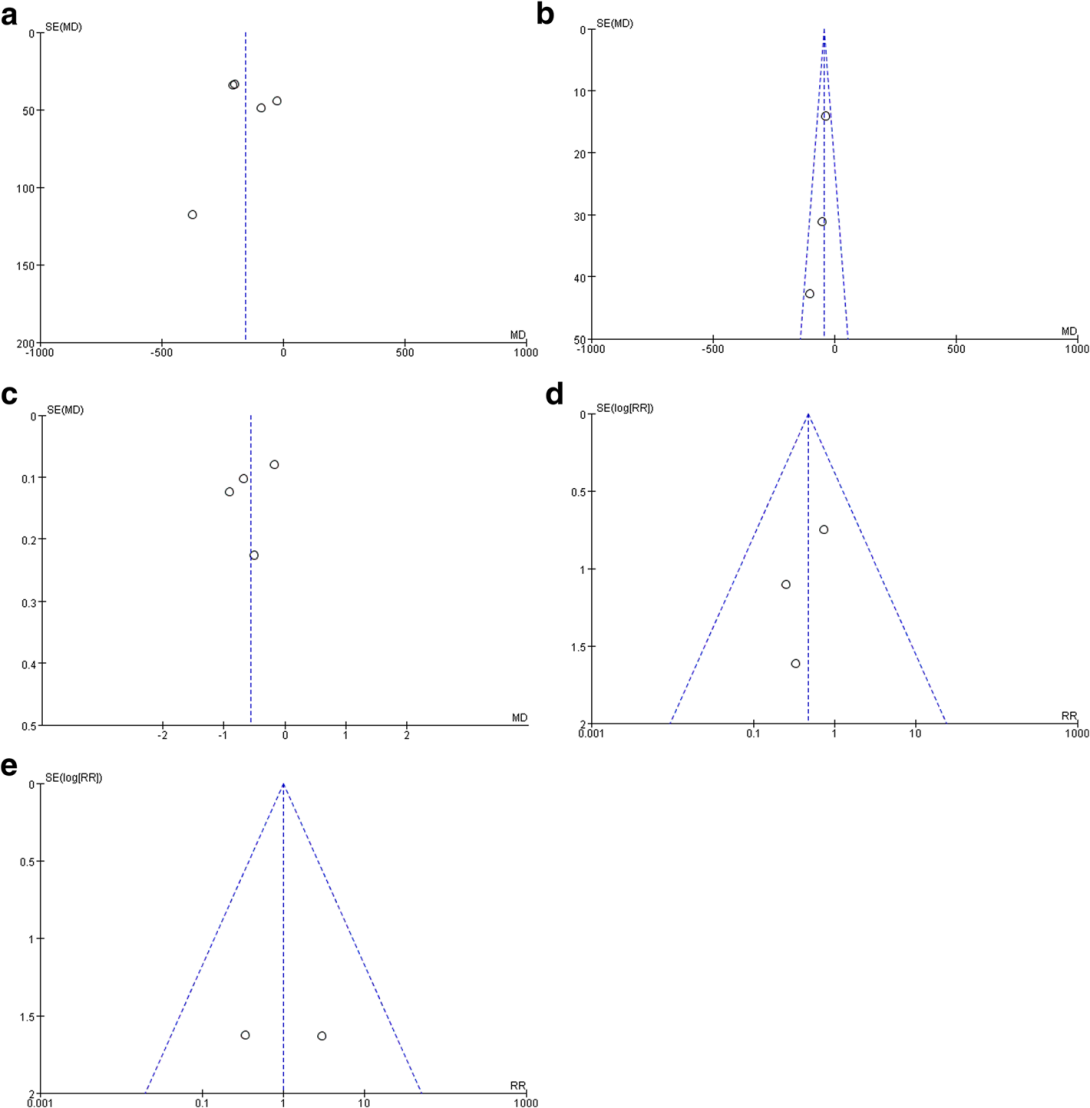
Funnel plot of total blood loss (**a**), drainage volume (**b**), maximum hemoglobin drop (**c**), transfusion requirements (**d**), and DVT (**e**)

Table 2 summarizes the methodological quality and the risk of bias of the included studies. All 6 trials were relatively well designed, and the modified Jadad scores showed that the quality of the 6 trials was high, of which there were 2 at least 4 points, there were 3 up to 7 points. The meta-analysis used independently funnel plots of total blood loss, drainage volume, maximum hemoglobin drop, transfusion requirements and DVT to assess publication bias; the plots were generally symmetrical and shown a lower publication bias (Fig. 2 A, B, C, D, E).

**Table 2:** Quality assessment and modified Jadad score of included 6 randomized controlled trials

Studies (years)	Random sequence generation	Allocation concealment	Blinding	Incomplete outcome data	Selective reporting	Other bias	Modified Jadad score
Huang (2014) [16]	Low	Low	Low	Low	Low	Low	8
Liu (2015) [19]	Unclear	Unclear	Unclear	Low	Low	Low	4
Jain (2016) [17]	Low	Low	Low	Low	Low	Low	7
Nielsen (2016) [22]	Low	Low	Low	Low	Low	Low	8
Lee (2017) [23]	Low	Low	Unclear	Low	Low	Low	6
Song (2017) [24]	Low	Low	Low	Low	Low	Low	7

Abbreviation: Low:low risk of bias; Unclear: unclear risk of bias; High: high risk of bias

### Meta-analysis of blood loss

**Fig. 3:**
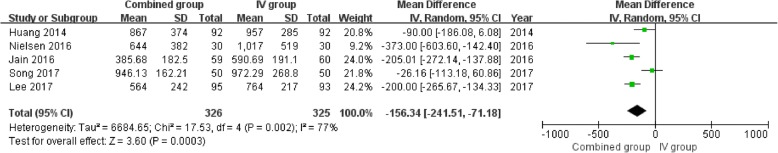
Forest plot analysis of total blood loss

A total of five studies [16, 17, 22–24] report relevant data regarding total blood loss (326 and 325 patients in the combined group and IV group, respectively). The outcome of meta-analysis indicates that total blood loss found in the combined group significantly reduced blood loss by a mean of 156.34 mL compared with the IV group (95% CI, − 241.51 to − 71.18; *P* = 0.0003) (Fig. 3). A random-effects model was used since there was significant heterogeneity among the studies (*P* = 0.002, I^2^ = 77%).

Subgroup analysis was performed base on the use of tourniquet or non-tourniquet, different topical (≤1.5 g or > 1.5 g) or number of IV TXA (single or ≥ 2 doses) of TXA. The outcome revealed that there was the significant difference between the two groups in terms of tourniquet or non-tourniquet, topical TXA dose or the number of IV TXA (Table 3).

**Table 3:** Results of meta-analysis and subgroup analyses of the included studies

Results (Combined vs. IV groups)	No.of studies / knee	P	Effect Size			
			MD/RR	95% CI	Heterogeneity p (I^2^)	Model
1. Total blood loss						
All studies	5/651	0.0003	−156.34	241.51 to −71.18	0.002 (77%)	Random
Tourniquet or Non-tourniquet						
Tourniquet	3/471	0.05	− 108.68	− 217.44 to 0.08	0.005 (81%)	Random
Non-tourniquet	2/179	0.0008	− 251.30	−398.40 to − 104.19	0.17 (47%)	Fixed
Topical TXA dose						
≤ 1.5 g	2/284	0.10	−54.93	−119.43 to 9.57	0.33 (0%)	Fixed
>1.5 g	3/367	< 0.00001	− 209.39	− 255.95 to − 162.83	0.36 (1%)	Fixed
Number of IV TXA						
Single dose	2/244	0.13	−210.91	− 485.30 to 63.47	0.03 (80%)	Random
≥ 2 doses	3/407	0.005	− 147.43	−251.16 to −43.69	0.002 (84%)	Random
2. Maximum hemoglobin drop						
All studies	4/591	0.003	−0.56	−0.93 to −0.19	< 0.00001 (90%)	Random
Tourniquet or Non-tourniquet						
Tourniquet	3/472	0.05	−0.52	−1.04 to 0.00	< 0.00001 (92%)	Random
Non-tourniquet	1/119	< 0.00001	−0.68	− 0.88 to − 0.48	–	–
Topical TXA dose						
≤ 1.5 g	2/284	0.006	−0.21	−0.35 to − 0.06	0.17 (47%)	Fixed
>1.5 g	2/307	< 0.00001	−0.78	−0.99 to − 0.56	0.17 (47%)	Fixed
Number of IV TXA						
Single dose	1/184	0.03	−0.17	−0.33 to − 0.01	–	–
≥ 2 doses	3/407	< 0.00001	−0.73	−0.93 to − 0.54	0.21 (36%)	Fixed
3. Transfusion requirements						
All studies	5/651	0.19	0.48	0.16 to 1.44	0.69 (0%)	Fixed
Tourniquet or Non-tourniquet						
Tourniquet	3/472	0.70	0.75	0.17 to 3.26	–	–
Non-tourniquet	2/179	0.16	0.28	0.05 to 1.64	0.89 (0%)	Fixed
Topical TXA dose						
≤ 1.5 g	3/344	0.50	0.64	0.17 to 2.37	0.65 (0%)	Fixed
>1.5 g	2/307	0.21	0.25	0.03 to 2.21	–	–
Number of IV TXA						
Single dose	2/244	0.50	0.64	0.17 to 2.37	0.65 (0%)	
≥ 2 doses	3/407	0.21	0.25	0.03 to 2.21	–	–
4. Drainage volume	3/334	0.0004	−43.54	−67.59 to −19.48	0.34 (7%)	Fixed
5. DVT	6/701	0.99	1.01	0.14 to 7.12	0.34 (0%)	Fixed

Abbreviation: RR, risk ratio; MDs, mean differences; IV, Intravenous; DVT, deep venous thrombosis

### 3.3Meta-analysis of drainage volume

**Fig. 4:**
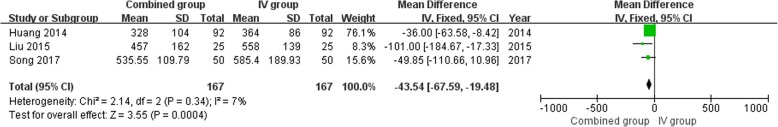
Forest plot analysis of drainage volume

A total of three studies [16, 24, 28] reported relevant data regarding drainage volume (167 and 167 patients in the combined group and IV group, respectively). The outcome of meta-analysis indicates that drainage volume found in the combined group significantly reduced drainage volume by a mean of 43.54 mL compared with the IV group (95% CI, − 67.59 to − 19.48; *P* = 0.0004) (Fig. 4). A fixed model was used since there was no significant heterogeneity among the studies (*P* = 0.34, I^2^ = 7%).

### Meta-analysis of maximum hemoglobin drop

**Fig. 5:**
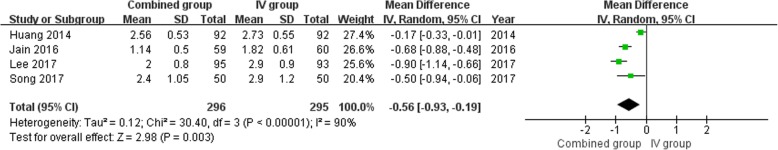
Forest plot analysis of maximum hemoglobin drop

A total of four studies [16, 17, 23, 24] reported relevant data regarding maximum hemoglobin drop (296 and 295 patients in the combined group and IV group, respectively). The outcome of meta-analysis indicates that maximum hemoglobin drop found in the combined group significantly reduced maximum hemoglobin drop by a mean of 0.56 g/dl compared with the IV group (95% CI, − 0.93 to − 0.19;*P* = 0.003) (Fig. 5). A random-effects model was used since there was significant heterogeneity among the studies (*P* <  0.00001, I^2^ = 90%). Similar results were received in subgroup analysis based on the application of tourniquet or non-tourniquet, topical TXA dose or the number of IV TXA (Table 4).

### Meta-analysis of transfusion requirements

**Fig. 6:**
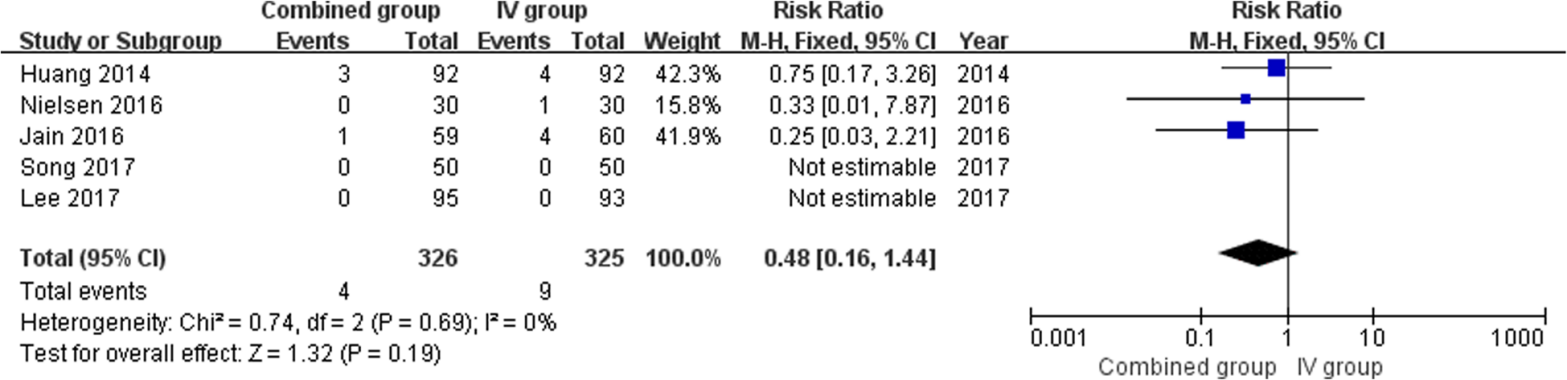
Forest plot analysis of transfusion requirements

A total of five studies [16, 17, 22–24] reported relevant data regarding transfusion requirements (326 and 325 patients in the combined group and IV group, respectively). The outcome of meta-analysis indicates that transfusion requirements found 4 of 326 patients in the combined group, compared to 9 of 325 patients in the IV group. The risk ratio (RR) shown there was no significant difference between the two group in the incidence of transfusion requirement (RR = 0.48, 95% CI, 0.16 to 1.44;*P* = 0.19) (Fig. 6). A Fixed-effects model was used since there was no significant heterogeneity among the studies (*P* = 0.69, I^2^ = 0%). Similar results were received from subgroup analysis based on the application of the tourniquet or non-tourniquet, topical dose and number of IV TXA (Table 4).

### Meta-analysis of DVT and PE

**Fig. 7:**
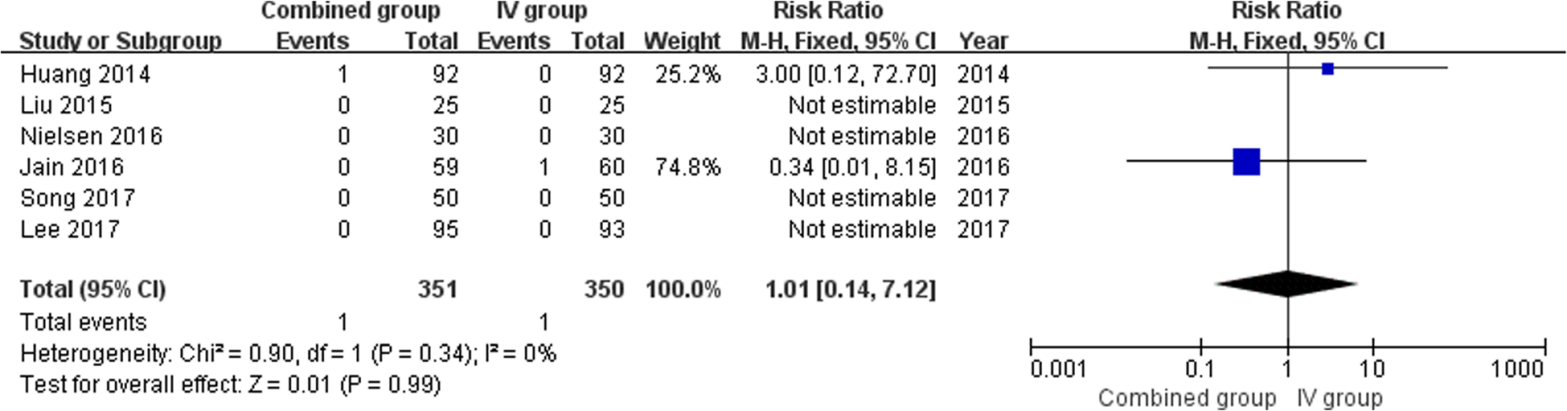
Forest plot analysis of DVT

A total of six studies [16, 17, 22–24, 28] reported relevant data regarding DVT (351 and 350 patients in the combined group and IV group, respectively). There was 1 in each of the two groups. Of which, 1 of 92 patients [16] who received combination intravenous and topical TXA developed DVT, and 1 of 60 patients [17] who received only intravenous TXA developed DVT (RR 1.01, 95% CI, 0.14 to 7.12;*P* = 0.99) (Fig. 7). All but 1 study [23] reported 1 PE found in the IVTXA (RR 0.33,95% CI, 0.01 to 7.91;*P* = 0.49). Thus, no significant differences were found in the two groups in terms of the incidence of DVT and PE. A fixed-effects model was used, since there was no significant heterogeneity among the studies (*P* = 0.34, I^2^ = 0%).

## Discussion

Tranexamic acid, an analog of the amino acid lysine, successfully reduces perioperative blood loss and transfusion requirements in primary TKA [5, 6, 29, 30]. TXA can be applied during the perioperative period either intravenously [13, 31], topically [8, 32], and orally [15, 33]. Recently, an increasing number of studies have focused on the issue that when compared with only intravenous or topical TXA, whether combination application of IV and topical TXA has additional benefits in primary TKA [16, 17, 22]. Thus, we performed the meta-analysis to assess the efficacy and safety of combined application versus intravenous application of TXA in TKA.

The main finding of this meta-analysis is that the combined application intravenous and topical of TXA can significantly reduce postoperative total blood loss, drainage volume, maximum hemoglobin drop compared to the application of intravenous TXA alone. Subgroup analyses showed that total blood loss was less in patients with non-tourniquet, topical TXA dose > 1.5 g and IV TXA ≥ 2 doses. No significant differences were found in the incidence of DVT and PE between the two groups.

The administration of IV TXA in TKA has been well established in a lot of literature. Akgül et al. [34] reported 20 mg/kg IV-TXA given before the skin incision in the primary TKA could decrease the total blood loss from 1166.42 mL to 634.03 mL and reduce the drainage volume from 640.74 mL to 311.11 mL. Pitta et al. [35] performed another retrospective study involving 610 patients during 4 years, they reported the administered IV of TXA resulted in a significant decrease by 9.4% in blood loss compared to the control group in TKA, and no significantly different was found in the incidence of DVT. Compared with the safety concerns with intravenous administration, topical TXA has been a growing interest to prevent bleeding. As previously reported, it was considered to less of 70% systemic absorption and thus may be a systemic alternative. Ishida et al. [36] conducted a randomized controlled trial that injected 2000 mg/20 mL topical TXA compared with a placebo group in TKA, the results revealed that postoperative decreasing in Hb level and knee joint swelling was significantly reduced in the TXA group compared to the control group. Recently, a new strategy of combined administration of TXA was explored considering the advantages of both methods. A randomized, double-blind, placebo-controlled trial [22] of 60 patients comparing patients who received combined IV and topical TXA or IV TXA in primary TKA found that combined administration results in significantly lower total blood loss and postoperative Hb level, while the incidence of DVT was similar between the two groups. Lee et al. [23] have also found the similar results that while there were no patients in any study group received an allogeneic transfusion, the combined group had lower total blood loss than the IV-only group.

The current meta-analysis shows that combined group could effectively reduce postoperative total blood loss by about 156.34 mL compared with IV TXA alone. Similarly, subgroup analysis suggested that there is also the significant difference between the two group in terms of the use of tourniquet or non-tourniquet, topical (≤1.5 g or > 1.5 g) and the number of IV TXA (single or ≥ 2 doses). Furthermore, the meta-analysis indicates that drainage volume found in the combined group significantly reduced drainage volume by a mean of 43.54 mL compared with the IV group. Thus, the combined administration of TXA could be a reasonable alternative to IV TXA alone for decreasing blood loss and drainage volume in primary TKA.

In our meta-analysis, the rate of transfusion requirements was slightly less for the combined group (1.2%) than for the IV TXA group (2.8%), but the difference was not statistically significant (RR 0.48, *P* = 0.19). Subgroup analyses showed similar results for the tourniquet or non-tourniquet and different topical or the number of IV TXA. Huang et al. [16] performed one RCT, 1.5 g topical TXA administered combined with 1.5 g IV-TXA, and there was no statistically significant difference regarding transfusion requirements between the two groups. Another RCT [23], 119 patients were randomized into combined group and IV group alone, the combined group provided better results than IV alone in total blood loss (590.69 ± 191.1mLvs. 385.68 ± 182.5 mL, *P* <  0.001), but no difference in blood transfusion rate (6.6% vs. 1.6%, *P* = 0.364). The result of our meta-analysis evaluating transfusion rates was consistent with these studies.

Thromboembolic prophylaxis methods were performed in all of the studies and as follow: low-molecular-weight heparin, aspirin, and enoxaparin. As Anderson et al. [37] reported in “N Engl J Med”, they found that among patients who received 5 days of rivaroxaban prophylaxis following TKA/THA, extended prophylaxis with aspirin was not significantly different from rivaroxaban in the prevention of DVT. Additionally, all thromboembolic events were detected by clinical symptomatic, routine Doppler ultrasound, or CT angiography. Finally, there was 1 in each of the two groups. The total rate of DVT was 0.03%. There was no difference in both two group (*P* = 0.99). 1 PE was found in the IV TXA group. Our findings are consistent with those of RCT [17, 23] or meta-analysis [38, 39] which found that no previous studies have reported increased rates of symptomatic DVT or PE with the combined administration of TXA in TKA.

The current study had the following strengths. First, all of the included studies have been well-designed and satisfied the defined eligibility criteria comparing the efficacy of combined TXA versus intravenous administration of TXA during TKA. Second, subgroup analysis was performed based on the use of the tourniquet or non-tourniquet and different topical or the number of IV TXA. The results showed that combined administration of TXA in TKA can effectively reduce total blood loss and maximum hemoglobin drop compared with IV TXA alone. Third, this study independently used funnel plots to assess publication bias, the plots were generally symmetrical and shown a lower publication bias. However, the meta-analysis also has limitations. First, the reported blood loss methods are not consistent, which may lead to deviations in the clinical outcome of blood loss. In addition, the method for estimating displacement is not detailed, so it cannot exclude the result deviation caused by human factors measurement. Second, the number of RCTs included was limited. Thus, more carefully and scientifically designed RCTs are needed in the future to further confirm and compare the different results. Third, due to the limited sample size of the study, our meta-analysis failed to extract adequate data pertaining to some items from these studies, such as function outcomes, patient satisfaction, and other complications, etc. Last, there was substantial heterogeneity in the meta-analysis of several outcomes, including total blood loss and drainage volume. However, taking into account the effects of the tourniquet and different topical TXA dose (≤1.5 g or > 1.5 g) or number of IV TXA (single or ≥ 2 doses), thus subgroup analysis was performed to decrease heterogeneity.

## Conclusions

This meta-analysis of currently available evidence indicates that combined administration of intravenous and topical TXA in primary TKA can significantly reduce total blood loss, drainage volume and maximum hemoglobin drop compared with IV TXA alone. The main finding, however, is that there is no significant difference in transfusion rates because this is the most clinically relevant point in this **meta-analysis.**

## Abbreviations

CI: Confidence interval
DVT: DVT
IV: Intravenous
MD: Mean differences
PE: Pulmonary embolism
RCT: Randomized controlled trials
RR: Risk ratio
SD: Standard deviation;
TA: Tranexamic acid
TKA: Total knee arthroplasty
TKR: Total knee replacement
TXA: Tranexamic acid
